# Women Who Smoke

**Published:** 1880-04

**Authors:** 


*Physician and Surgeon.*

### WOMEN WHO SMOKE.

The Society of Public Medicine and Professional Hygiene of Paris, at its session on the 24th of December, 1879, discussed the above topic. During the discussion the following facts were elicited.

Midwives who have attended the women that work in the tobacco factories situated in the *rue* Jean Nicot have observed that these women are diseased during gestation; that tobacco suppresses the secretion of milk which is less clear and rich than normal; that children nourished on such milk are not healthy and die in great numbers. In the same locality, tobacco has the reputation of causing persistent abortions, and the working women who can afford it, stop work, do not go to the factories when they become enceinte. One of the members of the society, M. Delaunay, stated that the children of the locality where the factory hands live are more difficult to raise, and that the mortality is greater among them than among other children of the same age.

Finally, tobacco effects the health of pregnant women and causes abortions. It also effects the health of the embryo which is born diseased; it diminishes the quantity and vitiates the quality of the milk, and in consequence retards the development of the child who often dies the victim of his mother's calling.

It is but just to mention that the doctors present disagreed on the influence of tobacco, one of them claiming that in the department Finistere where women smoke, abortions are no more numerous than in other parts of France. The discussion is to be resumed and probably the matter will be decided by more careful and exhaustive investigations.

